# The mosaic of experience: How individual differences in attention and working memory shape event segmentation

**DOI:** 10.1186/s41235-026-00729-7

**Published:** 2026-04-15

**Authors:** Berna Güler, Eren Günseli

**Affiliations:** Sabancı University, Istanbul, Turkey

**Keywords:** Event segmentation, Individual differences, Attentional control, Working memory

## Abstract

Episodic memories, although experienced as continuous, are structured into discrete events, a process supported by working memory (WM) and attentional control. Yet, the causal contributions of these mechanisms remain underspecified. This review synthesizes behavioral, cognitive, and neural findings from healthy aging and three cognitive profiles with known WM and attentional control impairments (attention-deficit/hyperactivity disorder, dyslexia, and obsessive–compulsive disorder) to clarify how these mechanisms shape event segmentation. Drawing on converging findings across these groups, we outline theoretically grounded expectations: aging may show preserved segmentation when semantic structure is firm but disruptions under interference and higher control demands; ADHD may exhibit coarser segmentation and reduced agreement due to attentional lapses and self-referential intrusions; dyslexia may show reduced fine-grained segmentation specifically for rapidly changing verbal events due to temporal-processing limits; and OCD may demonstrate schema-driven, idiosyncratic boundary placement under threat-relevant contexts. Integrating these findings, we propose a mechanism-centered framework in which segmentation arises from the interaction of WM constraints, attentional control dynamics, and schema/contextual modulation. This framework refines prediction–error–based accounts and generates testable hypotheses for future experimental work.

## Significance statement

Humans experience life as a continuous stream, yet our memories are stored as separate events, a phenomenon called event segmentation. This review examines how two core cognitive systems—attention and working memory—shape this segmentation process in aging, attention-deficit/hyperactivity disorder, dyslexia, and obsessive-compulsive disorder. Studying these populations helps reveal which specific attentional and memory processes support event segmentation and how disruptions to these systems alter it. Clarifying these mechanisms both improves theories of how healthy people structure experience in memory and highlights potential targets for interventions and rehabilitation in groups with attentional or memory difficulties. The experimental predictions offered here provide concrete directions for future research aimed at identifying the cognitive and neural processes that organize continuous experience into meaningful events.

## Introduction

Even though we experience the world as a continuous flow of information, these experiences are segmented into distinct memory units, a process called *event segmentation*. Segmentation follows a hierarchical structure from fine-grained memory units, such as surfing, swimming, and sunbathing, to coarser-grained events, such as the beach event (Richmond et al., 2017). Event boundaries mark the transitions between these units, typically occurring at contextual shifts such as changes in location, emotion, or motivational states (Güler et al., 2025; McClay et al., 2023; Shin & DuBrow, 2021; Wang & Egner, 2022). Segmentation shapes memory organization: items within the same event are perceived as closer in time and recalled with greater temporal order accuracy than items that across event boundaries (DuBrow & Davachi, 2013; Ezzyat & Davachi, 2014; Heusser et al., 2018).

Event segmentation has been theoretically linked to working memory (WM) and attentional control, which are thought to support the construction and updating of event models (Bailey et al., 2017; Faber et al., 2018; Radvansky & Zacks, 2017; Zacks, 2020). However, direct causal tests of attention and WM in event segmentation remain scarce, leaving their precise roles in event segmentation unclear. Given this scarcity, comparing groups with dissociable attentional control and WM constraints could offer a mechanism-based understanding beyond descriptive accounts.

Event segmentation, which shows a high inter-individual agreement (Newtson, 1973; Speer et al., 2003), can also be significantly affected by inter-individual differences (Kurby & Zacks, 2011; Ryan & Rogers, 2021; Ryan et al., 2023; Sava-Segal et al., 2023; Swallow & Wang, 2020; Zacks & Sargent, 2010; Zacks et al., 2006). Such differences are especially pronounced in aging and specific profiles, where deficits in WM and attentional control are common (Ryan & Rogers, 2021; Zacks et al., 2006). These populations, therefore, allow us to investigate how limitations in these core cognitive resources affect segmentation processes.

By integrating findings from diverse cognitive profiles and healthy aging, this review aims to clarify the roles of WM and attentional control in event segmentation and to demonstrate how examining these processes in different cognitive profiles can provide insight into the mechanisms supporting episodic memory organization. Accordingly, we selected attention deficit hyperactivity disorder (ADHD; Mostert et al., 2015), dyslexia (Alt et al., 2022), and obsessive–compulsive disorder (OCD; Perna et al., 2019). Because (i) these profiles show distinct deficits in attentional control and WM, (ii) they remain relatively underexamined from an event-segmentation perspective despite recent empirical work and reviews have highlighted them for further investigation, and (iii) together they allow contrastive and mechanism-driven predictions. In that sense, we treat these profiles as convergent tests of how attentional control and WM constraints shape event formation. Relevant studies were identified through searches in PsycINFO, PubMed, Scopus, and Web of Science using combinations of terms related to event segmentation, working memory, attentional control, aging, and the cognitive profiles reviewed.

## The role of attentional control and WM on event segmentation

Event segmentation involves encoding the ongoing activity (e.g., maintaining an event model), updating event items in WM, and associating event items (Radvansky, 2012; Zacks, 2020). When the current event model fails to predict upcoming information, as reflected in event transitions, WM replaces the existing representation with a new one (Kurby & Zacks, 2018; Ongchoco & Scholl, 2019; Sargent et al., 2013). WM capacity is important in this sense for segmentation, as individuals with higher WM capacity tend to exhibit normative segmentation agreement, which is linked to better recall of event details (Bailey et al., 2013a, 2013b; Sargent et al., 2013). This finding was preserved even after controlling for age, education, perceptual speed, episodic memory, and general knowledge (Sargent et al., 2013). In that sense, WM capacity is important for segmentation, as individuals hold current-event representations and bind within-event items to one another and to previous experiences (Hahamy et al., 2023; Zacks, 2020).

Recently, two possible models regarding the specific contribution of WM in event segmentation have been proposed: accumulation and reactivation (Güler et al., 2024). The accumulation model suggests that within-event items are accumulated in WM during encoding and reactivated at boundaries, and then they are transferred to long-term memory. On the other hand, the reactivation model suggests that within-event items are individually transferred from WM to long-term memory during encoding, and all items are reactivated as an event in WM when a boundary is encountered. It has also been discussed that these models can work alternately or together, depending on the needs of the event. EEG findings further suggest that WM supports event segmentation primarily via boundary-triggered reactivation (Güler et al., 2025), providing a perspective on how WM deals with the information during event comprehension.

**Fig. 1:**
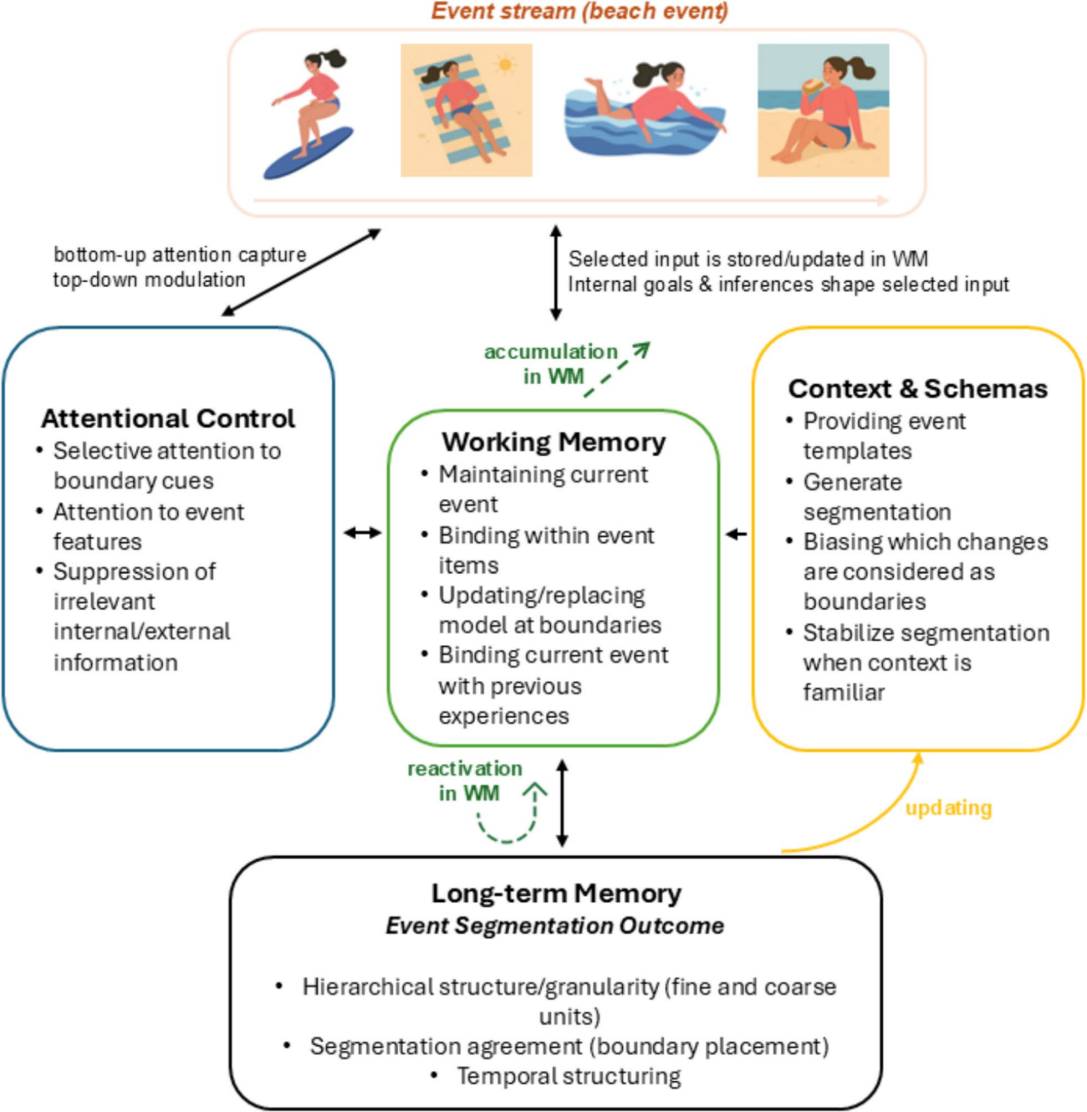
Proposed mechanistic model of how attentional control, working memory, and context jointly shape event segmentation. Perceptual information is captured bottom-up by salient changes and guided top-down by internal goals, and the resulting event representations are stored or updated in working memory. Attentional control and working memory interact bidirectionally to guide boundary detection and the updating or replacement of event representations. Context and schemas influence segmentation indirectly, by shaping top-down attentional priorities and constraining how incoming information is interpreted in WM. Together, these mechanisms give rise to long-term event-memory outcomes, including hierarchical structure, segmentation agreement, and temporal organization

Attentional control also plays a critical role in segmentation by allocating resources to event items, especially to transitions, as measured by increased dwell time (response time) at boundaries (Hard et al., 2011; Radvansky & Zacks, 2017). This might be beneficial, as event transitions carry information about important changes and upcoming representations (Hard et al., 2019). Attentional allocation decreases after the coarse-grained items (boundaries), suggesting that the updated event representation is more predictable and that higher asttentional allocation is not required to comprehend the event (Kosie & Baldwin, 2019) (Fig. 1).

## Individual differences in event segmentation

Individual differences in event segmentation provide an opportunity to understand how episodic memory is encoded and retrieved. While segmentation indicates broad consistency across people, significant variability arises in how structural changes in events are detected and represented. Neuroimaging studies suggest that fine-grained events are rapidly captured in low-level sensory regions (e.g., primary visual and sensory cortices). In contrast, coarse-grained events unfold more slowly in higher-order areas, such as the posterior medial cortex (Baldassano et al., 2017). Importantly, higher-level brain regions also show greater individual variability in detecting event boundaries, while activity in the sensory areas tends to be more synchronous across individuals (Sava-Segal et al., 2023). This variability may reflect differences in prior experiences, interpretive frameworks, or processing strategies. Consistent with this view, the involvement of higher-order areas connected to the default-mode network—known for its role in social cognition, representing situation models, autobiographical memory, and internal reflection—suggests that individual differences in segmentation may be partly shaped by how people draw on internally driven processes to structure experience (Barnett & Bellana, 2025; Yeshurun et al., 2021).

Research on clinical populations has identified common impairments in event segmentation. The first relates to segmentation agreement, measured by the similarity between an individual’s segmentation and the group norm (Eisenberg et al., 2016). Individuals with PTSD (Eisenberg et al., 2016, 2023; Pitts et al., 2022a, 2022b), forms of dementia (Alzheimer’s and Parkinson’s; (Bailey et al., 2013a, 2013b; Wyrobnik et al., 2022; Zacks et al., 2006), and traumatic brain injury (Zacks et al., 2016) tend to show reduced segmentation agreement compared to young or healthy older adults. A second common impairment concerns the hierarchical components of segmentation. For example, individuals with PTSD recall fewer fine-grained event details (Pitts et al., 2022a, 2022b, 2023), whereas those with traumatic brain injury or frontal lobe damage struggle more with coarse-grained segmentation compared to individuals with lower symptom severity or healthy controls (Zacks et al., 2016; Zalla et al., 2003). Such dysfunctions are often linked to deficits in predictive processing, memory functions, attentional processes, and cognitive control, and are associated with disruptions in regions such as the medial frontal cortex, amygdala, anterior cingulate cortex (Britton et al., 2005), medial temporal lobe (Bailey et al., 2013a, 2013b), and prefrontal cortex (PFC) (Zacks et al., 2016; Zalla et al., 2003).

Beyond these commonalities, distinct cognitive processes also appear to contribute to different dysfunctions. For example, dementia impairs the ability to segment memories into meaningful chunks (Bailey et al., 2013a, 2013b) and is associated with difficulties naming specific events and maintaining temporal sequence accuracy (Kokje et al., 2022; Wyrobnik et al., 2022). Individuals with PTSD, on the other hand, show difficulty predicting upcoming experiences and allocate less attention to objects encountered near the event transitions (Eisenberg et al., 2023). The authors discussed this within a conceptual framework proposing that hypervigilance may weaken predictive processing even in everyday, neutral contexts.

Taken together, these findings show that dysfunctions in attentional control and memory processes disrupt the hierarchical structuring of continuous experience into events. An intriguing extension is that similar variations in event segmentation are also observed in the context of healthy aging, raising the question of which factors most strongly contribute to these differences.

### How does the segmentation of memories change with aging?

To contextualize how age-related changes manifest in event segmentation, it is important to clarify how aging is defined across the reviewed studies below. Older adults are defined relatively consistently as individuals between 60 and 85 years of age, compared with young adults aged 18–30. Episodic memory typically deteriorates with aging, and impairments are most commonly observed in temporal order memory, source knowledge, and spatial location information (Berna et al., 2012; Craik & Henry, 2023; Old & Naveh-Benjamin, 2008). Mirroring these decrements, segmentation ability has also been observed to decline (Kurby & Zacks, 2011, 2012, 2018; Zacks et al., 2006). Research has shown that older adults are less able to track high-level goals at the coarse-grained level and report fewer fine-grained segments (Kurby et al., 2014; Kurby & Zacks, 2019; but see Magliano et al., 2012; Smith et al., 2020). They also perform worse than younger adults in event memory and in the temporal order of events(Kurby & Zacks, 2011, 2018; Seewald et al., 2018; Zacks et al., 2006). Studies have primarily associated aging-related segmentation impairments with declines in attentional control and memory processes, including reduced sustained attention, reduced WM, and difficulties in set shifting. These cognitive declines have been associated with inadequate representation of events, cognitive control biases, and weaker episodic encoding (Skrotzki et al., 2022; Smith et al., 2024).

However, preserved segmentation patterns are also evident in older adults. For example, some studies show comparable segmentation agreement relative to younger adults (Kurby et al., 2014; Reagh et al., 2020; Sargent et al., 2013; Smith et al., 2024). Moreover, older adults can generate, update, and recall event details during narrative comprehension (Radvansky & Dijkstra, 2007). When cued with event transitions, they benefit equally from these cues as younger adults, showing improved recall after a short delay (Gold et al., 2017). They also exhibit the same pattern of deteriorated recognition across event boundaries (Radvansky et al., 2015) and similar temporal compression within an event as younger adults (Folville et al., 2020). This opens an intriguing area for discussing the role of individual differences in event segmentation and how segmentation is affected by aging.

How can these mixed findings be reconciled, and through which mechanisms do older adults compensate for episodic memory declines when it comes to segmentation? We propose that some of the effects attributed to worsening event structure may instead reflect a shift toward increased reliance on semantic knowledge and event schemas. Below, we outline two mechanisms that support this interpretation.

First, older adults can perform event segmentation at levels comparable to younger adults under conditions that provide meaningful semantic structure, and this may be explained by increased reliance on semantic knowledge and event schemas. Event schemas help structure experience into meaningful units, and greater semantic knowledge predicts improved event comprehension and memory (Zacks & Tversky, 2001; Zacks et al., 2006). With age, situation models remain relatively preserved (Radvansky & Dijkstra, 2007), while semantic knowledge increases (Salthouse, 2004, 2019; Verhaeghen, 2003), providing a stronger scaffold for event comprehension and retrieval (Rusted et al., 2000). Consistent with this account, older adults perform segmentation at levels comparable to younger adults when the semantic context is familiar (Pitts et al., 2022a, 2022b; Smith et al., 2020, 2022). Thus, reduced fine-grained segmentation in aging may not necessarily reflect impaired event model construction (Zacks & Sargent, 2010), but rather a stronger reliance on semantic knowledge that prioritizes higher-level meaningful units and changes the event structure. Converging evidence from domain-expertise research shows that experts rely less on fine-grained, moment-to-moment transitions and more integrated units that reflect higher-level goals or schemas (Bläsing, 2015; Feller et al., 2023; Newberry et al., 2021), with segmentation advantages emerging only at coarse levels (Newberry et al., 2021).

Second, older adults may rely more on top-down attentional control when comprehending an event to compensate for reduced bottom-up processing (Madden et al., 2007), a process that is more susceptible to schematic influence (Sweegers et al., 2015). This top-down, schema-guided mode of processing may reduce the hierarchical differentiation of event representations and produce larger, more unitized event chunks relative to younger adults.

These observations offer an alternative to the view that older adults cannot perceptually identify event structure or maintain/update event models (Kurby & Zacks, 2011; Kurby et al., 2014; Zacks & Sargent, 2010). Instead, they suggest that at least some age-related patterns reflect schema-based strategies that privilege coarse-grained organization.

However, the semantic-based account is unlikely to generalize to all age-related segmentation deficits. When events are unfamiliar, weakly structured, or require sustained maintenance across contextual shifts, older adults exhibit increased vulnerability to interference (Kurby & Zacks, 2018; Reagh et al., 2020). With age, the number and strength of associative links surrounding an event increase, which can heighten competition among overlapping long-term memory traces (Grilli & Sheldon, 2022). Such interference may arise both at encoding (e.g., proactive interference from previously activated schemas) and at retrieval (e.g., overlap among semantically related traces). These interferences can destabilize the construction of a coherent event hierarchy, leading to less consistent coarse-grained boundaries and fewer fine-grained segments.

A complementary, boundary-triggered interference emerges when an event boundary introduces a contextual shift between the cue and the probe (Skrotzki et al., 2024). Because older adults rely more on reactive control, they are less likely to keep the cue active in WM across a boundary (Paxton et al., 2008). When the probe appears at the boundary, they must retrieve the cue from long-term memory, where it competes with overlapping traces, making reactivation less reliable. Converging behavioral evidence suggests that, under reactive demands, interference costs reflect the inefficient suppression of distractor-driven response activation. In contrast, for proactive control, context-based adjustments remain comparable to those of younger adults (Xiang et al., 2016). Together, long-term trace competition and boundary-induced reactive demands interact to increase interference in aging, particularly when semantic scaffolds offer limited support.

Taken together, these findings suggest a dual-pathway account of event segmentation in aging. On the one hand, a schema-guided pathway can support segmentation performance when events are meaningful, familiar, or semantically interpretable, leading older adults to rely on top-down semantic scaffolds to organize experience into coarse, integrated units. On the other hand, a control-dependent pathway appears more vulnerable to age-related declines when segmentation demands maintaining fine-grained, perceptual, or context-specific details across time. Under such conditions, particularly when semantic support is weak, ambiguous, or unavailable, older adults show increased susceptibility to interference, which may disrupt the maintenance, updating, or retrieval of fine-grained event features. Thus, age-related segmentation outcomes may reflect the relative balance between semantic-scaffolded organization and control-dependent, interference-prone processing, rather than a uniform deficit in event model construction.

#### Experimental predictions for the role of attentional control in aging and segmentation

Studies on individual differences in event segmentation generally highlight the roles of sustained attention and WM (Radvansky, 2017). However, because these roles have mostly been inferred from impairments in specific groups, the underlying processes have rarely been directly manipulated and tested. To our knowledge, one exception is Pradhan and Kumar (2022), who experimentally reduced attentional resources in young adults by adding a go/no-go task during encoding and observed a reduced boundary advantage relative to simple probe detection. Yet, this manipulation did not assess how attentional load impacts segmentation agreement or the hierarchical structure of events.

A straightforward next step would be to randomize high-performing young adults (baseline: good agreement and clear hierarchical structuring) to either (i) an attentional-load condition (concurrent go/no-go during encoding) or (ii) a low-load control (simple probe detection) while they segment the same materials. If attentional control is causal for segmentation, the load group should show weaker boundary advantage, lower agreement with the group norm, and poor hierarchical structure. Such a pattern would inform us about the profile often observed in older adults and would strengthen the claim that attentional constraints influence segmentation rather than global perceptual deficits.

Moreover, because the aging framework proposed here assumes not only impairment under cognitive load but also compensatory benefit from semantic scaffolding, a complementary manipulation would involve varying the semantic richness or familiarity of event sequences. This would allow testing whether semantic context mitigates attentional-load costs, as predicted by the proposed dual-pathway account.

Together, the aging evidence and experimental predictions above specify how attentional control and semantic scaffolding may jointly shape event segmentation. The following section extends this framework to cognitive profiles with documented deficits in attentional control and WM, examining whether they exhibit convergent or dissociable patterns of segmentation.

## Individual differences in cognitive profiles and event segmentation

Event segmentation has been studied across a range of conditions, and WM and attention have often been claimed to be critical for segmentation, yet the role of attentional control and WM in shaping individual differences remains only partially understood. Building on our framework above, we focus on four cognitive profiles with deficits in attention and WM (see Table 1). For each cognitive profile, we (i) summarize the cognitive phenotype relevant to attentional control and WM, (ii) summarize the related findings and derive predictions for segmentation patterns (e.g., agreement, granularity, boundary sensitivity), and (iii) highlight open questions for future research.

**Table 1:** Mechanistic mapping of how distinct cognitive constraints (WM, attentional control, schema bias, temporal processing) are expected to shape event segmentation patterns across aging and cognitive profiles

Group	Core cognitive constraints	WM/attentional mechanism	Expected segmentation pattern
Aging	•Interference susceptibility •Reduced WM processes •Sustained attention problems •Reliance on reactive control	•Semantic scaffolding supports event structure •Boundary updating vulnerable under load	•Comparable to controls when the semantic contest is rich •Coarser and less consistent segmentation under high cognitive load or unfamiliar events
ADHD	•Sustained attention deficits •Executive dysfunction •Intrusion control failures	•Event model updating disrupted by self-referential/default mode network intrusions •Unstable top-down control	•Over-segmentation at coarse level •Lower agreement with group norms, especially under self-referential or emotionally evocative content
Dyslexia	•Impaired temporal resolution for rapid verbal input •Verbal WM load	•Reduced temporal sampling fidelity •Increased temporal demand for rapidly changing verbal cues	•Fewer fine-grained segmentation for rapidly changing verbal events •Segmentation ability comparable to controls for visuospatial or temporally regular input
OCD	•Inhibition deficit •Threat/uncertainty-driven schema bias	•Schema-congruent attentional capture •WM load from checking/monitoring	•Idiosyncratic, schema-consistent boundary placement •Reduced agreement when context lacks stability/neutrality

### Attention deficit hyperactivity disorder (ADHD)

#### Cognitive profile

ADHD is a neurodevelopmental disorder marked by deficits in attentional control (selective, divided, sustained), set shifting, processing speed, immediate/delayed recall, WM, and emotion regulation (Alderson et al., 2013; Butzbach et al., 2019; Huang-Pollock & Karalunas, 2010; Karalunas & Huang-Pollock, 2013; Kasper et al., 2012; Martinussen et al., 2005; Mowinckel et al., 2015; Tucha et al., 2008; Weigard & Huang-Pollock, 2017). Hyperactivity tends to decline with age, whereas attentional and executive problems are more persistent into adulthood (Adler et al., 2017; Barnett et al., 2001; Biederman, 2000). Neurocognitively, ADHD has been linked to altered functioning in PFC and dorsal attention systems, and insufficient default mode network (DMN) suppression during task engagement (Cortese et al., 2012; Fair et al., 2010; Hart et al., 2012; Martel et al., 2007; Uddin et al., 2008; Yasumura et al., 2019). These characteristics may increase cognitive load during continuous event processing and reduce the availability of resources needed for sustained model updating or within-event binding.

#### ADHD and event segmentation

Framing ADHD within event segmentation clarifies how event models are generated and updated under attentional dysregulation. To date, relatively few studies have examined event segmentation in ADHD. In these studies, participants segmented video clips into meaningful parts, either at coarse or fine-grained levels. Individuals with ADHD show preserved fine-grained segmentation but tend to mark more coarse-grained events than healthy controls (Ryan & Rogers, 2021; Ryan et al., 2023). One interpretation is that ADHD involves more frequent prediction errors and difficulties maintaining and updating event models over time.

An alternative account emphasizes contextual factors beyond prediction error in event segmentation, including internal motivations and self-referential processes (Güler et al., 2025; Shin & DuBrow, 2021). The DMN, which is associated with self-referential processing and the representation of situation models, has been linked to attentional dysregulation in ADHD (Barnett & Bellana, 2025; Buckner et al., 2008; Sonuga-Barke & Castellanos, 2007; Uddin et al., 2008). Asynchrony or insufficient suppression of DMN activity during effortful cognition can promote periodic attentional intrusions that compete with task-relevant representations. Related work indicates altered functional coupling within DMN during self-referential processing in ADHD (Van Buuren et al., 2010). In this view, fluctuations in sustained attention combined with DMN asynchrony reduce top-down control over what is attended within an unfolding event, biasing segmentation toward less hierarchically aligned memory units (Götting et al., 2017).

Comorbidity and social cognition provide an additional perspective. Compared to ADHD-only, ADHD with co-occurring anxiety shows lower segmentation agreement; moreover, greater segmentation frequency (over-segmentation) covaries with inattention/hyperactivity and poorer social skills (Ryan et al., 2022, 2023). Adult ADHD studies also report vulnerabilities in emotion processing, with mixed results for theory of mind (Morellini et al., 2022). A unifying interpretation is that attentional dysregulation (including mind-wandering) increases intrusions during event segmentation, leading to misaligned boundaries and downstream social difficulties. A mechanistic possibility is that insufficient task-related suppression of the DMN could increase such intrusions (Götting et al., 2017; Van Buuren et al., 2010). However, direct links between DMN dynamics and social segmentation in adult ADHD remain untested.

#### Experimental predictions for the role of attentional control and WM in ADHD

Individuals with ADHD are more susceptible to self-referential intrusions and mind-wandering (Ahmed et al., 2024; Van Buuren et al., 2010). Using neutral materials in which boundaries are defined by exogenous contextual shifts (e.g., object-category) should clarify whether coarse over-segmentation reflects difficulty with prediction or updating, or an inability to guide self-referential content. If, under neutral materials, segmentation agreement increases and the number of coarse boundaries decreases in ADHD (with minimal change in controls), this would suggest that intrusions of self-referential processing are driving previous findings, rather than indicating a global segmentation deficit.

Although studies have found that the determination of event boundaries varies in the healthy population during learning (e.g., Ryan et al., 2021, 2022, 2023), the encoded memory units have not been evaluated to identify differences in the structured events underlying long-term memory and recall processes. To address this gap, commonly employed assessments such as temporal order and temporal distance judgments (van de Ven et al., 2021; Wang & Egner, 2022; Wen & Egner, 2022) could be used to validate an increased number of coarse-grained events observed in individuals with ADHD. If the effect of the event boundaries specified during learning is not reflected in the encoded memory units in later memory tests, it can be said that event segmentation in people with ADHD differs between encoding and retrieval.

### Dyslexia

#### Cognitive profile

Although dyslexia is primarily characterized by phonological and orthographic processing difficulties (Georgiou et al., 2012; Ramus & Szenkovits, 2008), converging evidence suggests associated constraints in verbal WM, temporal processing, and attentional control, particularly for time-compressed or multi-modal input (Fostick & Revah, 2018; Harrar et al., 2014; Irak et al., 2019; Isaki et al., 2008; Litt et al., 2019; Menghini et al., 2011; Stein, 2014; Toffalini et al., 2018). One proposal is that WM plays a more prominent role while reading skills are not yet automatized in childhood, with its overt contribution appearing to decrease as reading becomes more automatic (Alloway et al., 2014); nevertheless, verbal WM deficits are still observed in young adults (Collis et al., 2013; Ghani & Gathercole, 2013).

At the neural level, evidence points to atypical functioning in fronto-parietal networks associated with executive control and WM, alongside alterations in left-hemisphere language circuits (Beneventi et al., 2010; Peterson & Pennington, 2012; Stein, 2014). These patterns suggest that dyslexia may influence how event information is accumulated, temporally integrated, and maintained, especially when verbal materials unfold quickly or require cross-modal coordination.

#### Dyslexia and event segmentation

Two lines of evidence, domain-specific memory deficits and temporal-processing limitations, offer complementary predictions for how dyslexia may change event formation. Episodic memory findings in dyslexia are heterogeneous and domain-specific, suggesting that memory formation varies across modalities. In the verbal domain, encoding difficulties have been attributed to inefficient rehearsal and encoding with relatively intact retrieval (Kramer et al., 2000). By contrast, reports for visual/visuospatial memory are inconsistent, with some showing impairments (Menghini et al., 2010), whereas others find intact visual episodic memory or even relative strengths in visuospatial recognition, especially for abstract, nonverbal materials (Hedenius et al., 2013; Kibby & Cohen, 2008; Li et al., 2009). Taken together, these patterns suggest that individuals with dyslexia may construct event representations differently across modalities, with potential consequences for the hierarchical structure of segmented events.

Temporal-processing limitations (Cantiani et al., 2013; Fostick & Revah, 2018; Rey et al., 2002; Ronconi et al., 2020) further imply that detecting boundary transitions may be more difficult, particularly when cues depend on phonemic or prosodic change rather than visuo-motor discontinuities. In line with this, individuals with dyslexia show reduced accuracy in temporally ordering and discriminating rapidly presented auditory–verbal stimuli (~ 140 ms) (Martino et al., 2001) and slower syntactic integration indexed by prolonged P600 latency (Cantiani et al., 2013; Sabisch et al., 2006) compared to controls. Importantly, performance improves when segment durations are lengthened (~ 280 ms) (Martino et al., 2001), suggesting that temporal sampling fidelity rather than global event-model impairment may constrain segmentation for fast, verbally cued boundaries. Thus, lower segmentation accuracy or agreement in verbally dense, rapidly changing events may reflect limitations in the temporal resolution of attention, which reduces the ability to detect and select boundary-relevant cues at the required pace, rather than a failure to construct hierarchical event models per se.

#### Experimental predictions for the processing of event segmentation in dyslexia

Evaluating event segmentation in dyslexia requires considering two constraints discussed above: (i) domain-specific verbal memory weaknesses and (ii) reduced temporal resolution for rapidly changing auditory–verbal input, both of which place demands on attentional sampling and WM integration. These constraints suggest that dyslexia may not show global impairment in segmentation, but rather modality- and temporal-dependent differences.

First, rapid verbal or phonological changes (e.g., phonemic shifts) should reduce segmentation agreement and decrease the number of boundaries relative to controls, because attentional sampling and WM integration cannot keep pace with input speed (Martino et al., 2001; Cantiani et al., 2013; Fostick et al., 2012). In contrast, visual or visuospatial materials, especially those lacking verbal load, should reduce or eliminate group differences, consistent with reports of preserved or relative strengths in nonverbal episodic memory (Hedenius et al., 2013; Kibby & Cohen, 2008; Li et al., 2009).

Second, if segmentation differences stem from temporal resolution demands (e.g., Menghini et al., 2010) rather than impaired model construction, then increasing temporal regularity (e.g., longer event segments, rhythmically spaced transitions, slowed narrative pace) should normalize segmentation agreement and hierarchical event structure.

Third, because verbal encoding difficulties are more pronounced than retrieval deficits (Kramer et al., 2000), dyslexia may show greater divergence from controls at encoding than at retrieval, resulting in weaker alignment in boundary detection at encoding but preserved temporal order and distance memory (e.g., within- vs across-event effects).

Together, these predictions emphasize that segmentation differences in dyslexia should emerge selectively under high verbal-temporal processing load, reflecting attentional resource and WM limits rather than a generalized deficit in forming hierarchical event representations.

### Obsessive–compulsive disorder (OCD)

#### Cognitive profile

OCD is characterized by intrusive obsessions and repetitive compulsions that are often driven by threat- and uncertainty-focused interpretations (Stein et al., 2019). Beyond these symptoms, individuals with OCD show deficits in episodic memory, particularly in organizational strategies at encoding and retrieval, and associative binding (Kalenzaga et al., 2020; Sawamura et al., 2005; Tuna et al., 2005; Vandborg et al., 2014). While they preserve the intentional forgetting ability, they recall to-be-remembered items less than controls (Konishi et al., 2011). In line with these, they are also less confident about their memory accuracy (Hermans et al., 2008). From a control-process perspective, OCD is associated with difficulties inhibiting task-irrelevant internal or external information, and schema-based attentional biases toward threat-relevant cues (De Geus et al., 2007; Tükel et al., 2012). In terms of WM, studies report reduced binding efficiency across modalities and lower short-term storage capacity (Harkin & Kessler, 2011; Jaafari et al., 2013; Nakao et al., 2009).

These control and WM constraints occur alongside frontostriatal circuit differences, including altered prefrontal activity and reduced volumes in the orbitofrontal cortex and basal ganglia (Batistuzzo et al., 2015; Friedlander & Desrocher, 2006; Menzies et al., 2008; Saxena & Rauch, 2022). Together, these features may disrupt event formation by increasing schema-driven interference, reducing suppression of irrelevant context, and limiting the binding resources required for coherent segmentation.

#### Obsessive–compulsive disorder and event segmentation

Direct tests of event segmentation in OCD are lacking, but Zacks and Sargent (2010) outlined possible mechanisms relevant to this process: (i) generated short-timescale prediction error leading to increased fine-grained segmentation, (ii) difficulty constructing coarse-grained structure because WM load from many fine units draws attention away from overarching goals in a given event, and (iii) distorted event schemas that bias how experiences are segmented.

Building on the broader OCD profile, an alternative view emphasizes context- and affect–driven segmentation rather than prediction error (Güler et al., 2025; Harris et al., 2025; Shin & DuBrow, 2021). Individuals with OCD often operate within rigid, threat- and uncertainty-oriented schemas in addition to attentional biases and reduced inhibition for task-irrelevant internal/external context and metacognitive lack of confidence (e.g., De Geus et al., 2007; Hermans et al., 2008; Oguz et al., 2019; Tükel et al., 2012). Such biases could shift boundary placement toward context-relevant events (e.g., cue checking, rule verification) that are idiosyncratic to the individual’s obsessions. On this account, segmentation agreement may deviate from group norms not because the perceptual signal is noisier to generate prediction error, but because schema-congruent priorities organize the hierarchy.

#### Experimental predictions for the processing of event segmentation in obsessive–compulsive disorder

Because event segmentation has not been systematically tested in OCD, two testable accounts can be considered. A prediction-error account (Zacks & Sargent, 2010) predicts denser fine-grained segmentation and difficulty building coarse structure when short-timescale discrepancies accumulate, due to WM overload from numerous subunits. A schema-guided/contextual stability account (e.g., Güler et al., 2025; Shin & DuBrow, 2021; Wang & Egner, 2022) predicts context-congruent boundary placement, especially for threat/uncertainty material, with performance normalizing when context is stable, because an externally defined stable structure reduces schema intrusions and allows WM and attentional resources to remain focused on event-relevant information. Accordingly, when viewing OCD-relevant vs neutral/stable stimuli, individuals with OCD should show (i) lower segmentation agreement and greater fine-grain density selectively for schema-relevant content, with these effects attenuated under neutral or externally defined boundaries, and (ii) longer boundary-locked response time at schema-relevant transitions, indexing attentional capture by contextual cues.

A second approach is to relate encoding to retrieval. If segmentation differences reflect a stable event structure, then memory tasks (temporal order and temporal distance judgments) should track the encoded boundaries for OCD-relevant materials. If, instead, differences arise mainly from online factors during encoding (e.g., underconfidence, checking, schema relevancy) that occupy WM and attentional resources, group gaps between control and individuals with OCD should be larger at encoding and reduced at test. To keep inferences specific, designs should include neutral vs OCD-relevant materials, manipulate context stability, and model anxiety comorbidity and WM as covariates, while matching perceptual demands across conditions.

## Conclusion

This review evaluated how attentional control and WM shape event segmentation across healthy aging and three cognitive profiles characterized by deficits in these processes: ADHD, dyslexia, and OCD. Synthesizing evidence across these groups points to three core claims. First, attentional control—including selection, sustained focus, shifting, and inhibition—influences boundary placement and event hierarchy, leading to misaligned or overly segmented events and reduced segmentation agreement. Second, characteristics of WM—capacity, timely processing, and domain-specific load—are closely linked to boundary placement and event hierarchy and may lead to misaligned or overly segmented events and reduced segmentation agreement. Third, semantic schemas and contextual stability can both buffer attentional/WM limitations by providing structure and bias segmentation by prioritizing schema-consistent boundaries.

These elements resulted in dissociable yet predictable patterns across groups. In aging, segmentation can look comparable to younger adults when semantic information is available, but boundary-triggered, reactive demands reveal greater interference, and proactive preparation reduces these costs. In ADHD, increased segmentation and reduced agreement indicate a potential outcome when self-referential content and mind-wandering are likely; neutral, exogenously defined boundaries can eliminate these effects. In individuals with dyslexia, deficits in temporal resolution are potentially associated with fewer fine-grained boundaries in irregular sequences, whereas relatively preserved segmentation is observed for visuospatial content and when temporal regularity is increased. In OCD, schema-guided segmentation (especially under threat/uncertainty) should produce idiosyncratic segmentation and weaker segmentation alignment, whereas stable, schema-neutral contexts should normalize performance. Theoretically, the profile patterns can be evaluated with a hybrid account in which segmentation arises from the interaction of WM processes, attention to change and boundary cues, and context/semantic priors.

In summary, exploring event segmentation in different cognitive profiles enhances our understanding of the role of WM and attentional control in the formation of episodic memories. This review offers a unified, mechanism-centered framework that highlights attentional control, WM capacity, and schema/context stability as processes that carry potential for further investigation to understand the memory hierarchy. It also suggests alternatives that refine the prediction-error account of event segmentation, emphasizing how WM, attentional control, and schemas may modulate or complement prediction-driven boundary detection across different profiles.

Taken together, the evidence across cognitive groups illustrates how disruptions in attention and WM reshape segmentation and supports a modeling framework in which segmentation behavior emerges from the interaction between (i) WM constraints, (ii) attentional control dynamics, and (iii) schema-based modulation, rather than from a uniform prediction-error process alone. Testing this interaction using standardized multi-modal segmentation paradigms will determine whether segmentation variability reflects capacity limitations, strategy differences, or both.

## Data Availability

Since the present study is a review paper, there are no participants or related data.
